# Recent advances in mRNA cancer vaccines: meeting challenges and embracing opportunities

**DOI:** 10.3389/fimmu.2023.1246682

**Published:** 2023-09-06

**Authors:** Bolin Wang, Jinli Pei, Shengnan Xu, Jie Liu, Jinming Yu

**Affiliations:** ^1^ Lung Cancer Center, West China Hospital, Sichuan University, Chengdu, China; ^2^ Department of Radiation Oncology and Shandong Provincial Key Laboratory of Radiation Oncology, Shandong Cancer Hospital and Institute, Shandong First Medical University and Shandong Academy of Medical Sciences, Jinan, Shandong, China; ^3^ Research Unit of Radiation Oncology, Chinese Academy of Medical Sciences, Jinan, Shandong, China

**Keywords:** mRNA cancer vaccines, cancer, cancer vaccine, personalized vaccine, cancer therapy, immunotherapy

## Abstract

Since the successful application of messenger RNA (mRNA) vaccines in preventing COVID-19, researchers have been striving to develop mRNA vaccines for clinical use, including those exploited for anti-tumor therapy. mRNA cancer vaccines have emerged as a promising novel approach to cancer immunotherapy, offering high specificity, better efficacy, and fewer side effects compared to traditional treatments. Multiple therapeutic mRNA cancer vaccines are being evaluated in preclinical and clinical trials, with promising early-phase results. However, the development of these vaccines faces various challenges, such as tumor heterogeneity, an immunosuppressive tumor microenvironment, and practical obstacles like vaccine administration methods and evaluation systems for clinical application. To address these challenges, we highlight recent advances from preclinical studies and clinical trials that provide insight into identifying obstacles associated with mRNA cancer vaccines and discuss potential strategies to overcome them. In the future, it is crucial to approach the development of mRNA cancer vaccines with caution and diligence while promoting innovation to overcome existing barriers. A delicate balance between opportunities and challenges will help guide the progress of this promising field towards its full potential.

## 1 Introduction

Cancer is one of the tremendous challenges to human health globally and the leading cause of death. Based on the most recent statistical report, about 5370 new occurrences, and 1670 deaths each day are expected during 2023 in the United States (1). Due to high morbidity and mortality rates, tremendous efforts have been devoted to the search for anticancer modalities (2, 3). Although advances have been made in traditional therapeutic methods, including surgery, chemotherapy, and radiotherapy, the reliable cure is still limited. In recent years, immunotherapy has become an essential focus for cancer treatment, and multiple immune checkpoint inhibitors (ICIs) are approved as a therapy for cancer (4, 5). ICIs with apparent efficacy and low side effects raise new hope for cancer patients and lead to an increased awareness of the influential role of the immune system in the success of anticancer therapy (5, 6). However, limited benefit populations, drug resistance, and high costs remain significant concerns for ICIs (7). Hence, there is an imminent need to look for novel and effective ways to activate the immune system to fight tumors.

Recently, the successful applications of COVID-19 global pandemic offered a great opportunity for messenger RNA (mRNA)vaccines in antitumor therapy (8–10). Therapeutic mRNA cancer vaccines as a novel immunotherapeutic strategy, which aims to kill tumor cells via invoking antitumor adaptive immune responses, have attracted great attention (11, 12). Specifically, therapeutic mRNA cancer vaccines encode the key components for the process of the immune response, such as tumor-specific antigens (TSAs), tumor-associated antigens (TAAs), and immune modulatory factors, thus promoting immune activation to perform antitumor functions (13, 14). Therapeutic mRNA cancer vaccines have been reported to provide stronger cellular or humoral immunity than traditional inactivated pathogen or protein-based vaccines (15). Not only that, it has the advantages of low cost, rapid development, safety and flexibility, and potent immunogenicity (14). Currently, multiple therapeutic mRNA cancer vaccines are being evaluated in phase I/II trial trials with promising early-phase results (14, 16). In view of therapeutic mRNA cancer vaccines are undergoing early stages of clinical development, it is crucial to more fully understand current status and challenges of therapeutic mRNA cancer vaccines.

In this review, we evaluate the benefits of therapeutic mRNA cancer vaccines while providing a succinct overview of their classification and mechanism. Particularly, we highlight recent advancements from preclinical studies and clinical trials that identify the obstacles associated with the development of mRNA cancer vaccines and discuss potential strategies to overcome them.

## 2 Advantages of mRNA cancer vaccines

mRNA vaccine has its origin in the 1990s when Wolff et al. found that mRNA could directly transfect muscle cells when injected *in vivo*, leading to the expression of the encoded related protein (16). However, its clinical application is restricted due to a lack of efficient synthesis, modifications, and delivery technologies (17). With recent breakthroughs and developments, the success of COVID-19 vaccines raised the hope for mRNA-based therapeutics for the treatment of various types of diseases, most notably in cancer.

**Figure 1:**
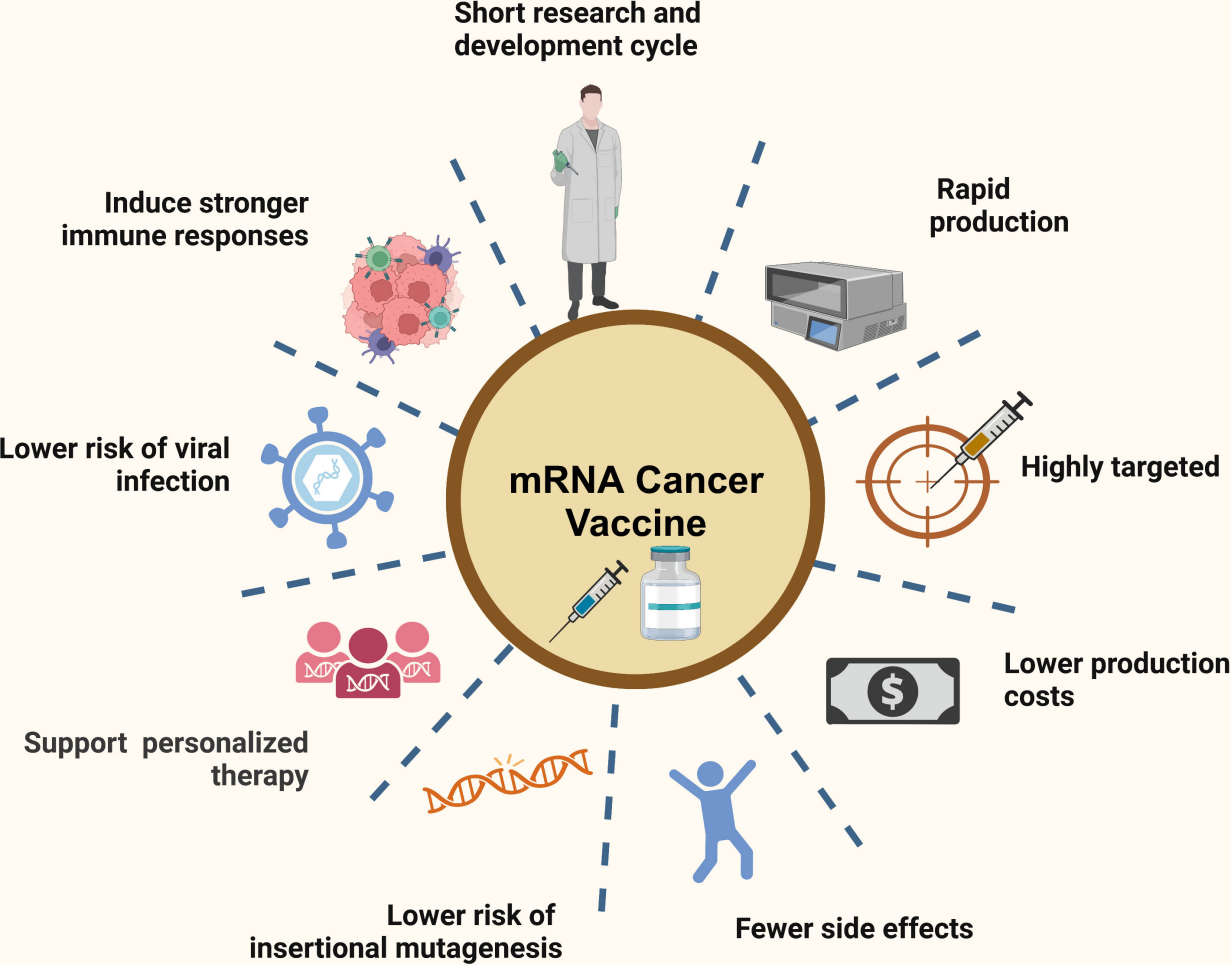
The advantages of mRNA vaccines in the context of cancer therapy.

The unique mechanism of action of the mRNA cancer vaccine has permitted the offer of advantages over conventional cancer therapy such as surgery, chemotherapy, and radiation therapy. **Figure 1** demonstrates the advantages of mRNA vaccines for the treatment of cancer. A primary advantage of mRNA cancer vaccine is potent immunogenicity, which supports strong humoral immune response and the cell-mediated immune response, thus exhibiting a strong anti-tumour effect (18, 19). For metastatic tumours, which are not easily cured by surgery, the mRNA cancer vaccine was found to be effective as it can provoke a systemic immune response (20, 21). Apart from the above, mRNA cancer vaccines can build and maintain long-term immunological memory making preventing tumour recurrence possible (16). Several of mRNA cancer vaccines has been shown to have potent therapeutic efficacy in preclinical cancer models for primary tumor and metastases (6, 21).

Another significant advantage of mRNA cancer vaccines is the ability to support personalized therapies, which can increase therapeutic efficacy and minimize side effects (18, 22). mRNA vaccines with highly versatile allow mRNA sequences to be easily tailored to encode personalized antigens or cytokines of interest to against cancer (11, 22). mRNA cancer vaccine as nucleic acid vaccine, which can be translated immediately after it enters the cytoplasm and entry into the nucleus is not required (23). In contrast to DNA vaccines, mRNA cancer vaccines could avoid the requirement of nuclear localization and the risk of insertional mutagenesis associated with DNA (18). Furthermore, mRNA cancer vaccines do not carry the risks of accidental infection, making it an essential safety issue (24, 25). Of particular significance, mRNA production is faster, more flexible and less expensive compared to many current vaccination strategies (26). For example, mRNA vaccination enables the fast and secure production of vaccines during pandemics such as SARS-CoV2 (27). Moreover, mRNA vaccines can be manufactured without encountering the complex, time-consuming challenges associated with plasmid DNA, viral vectors, or recombinant proteins (16).

**Figure 2:**
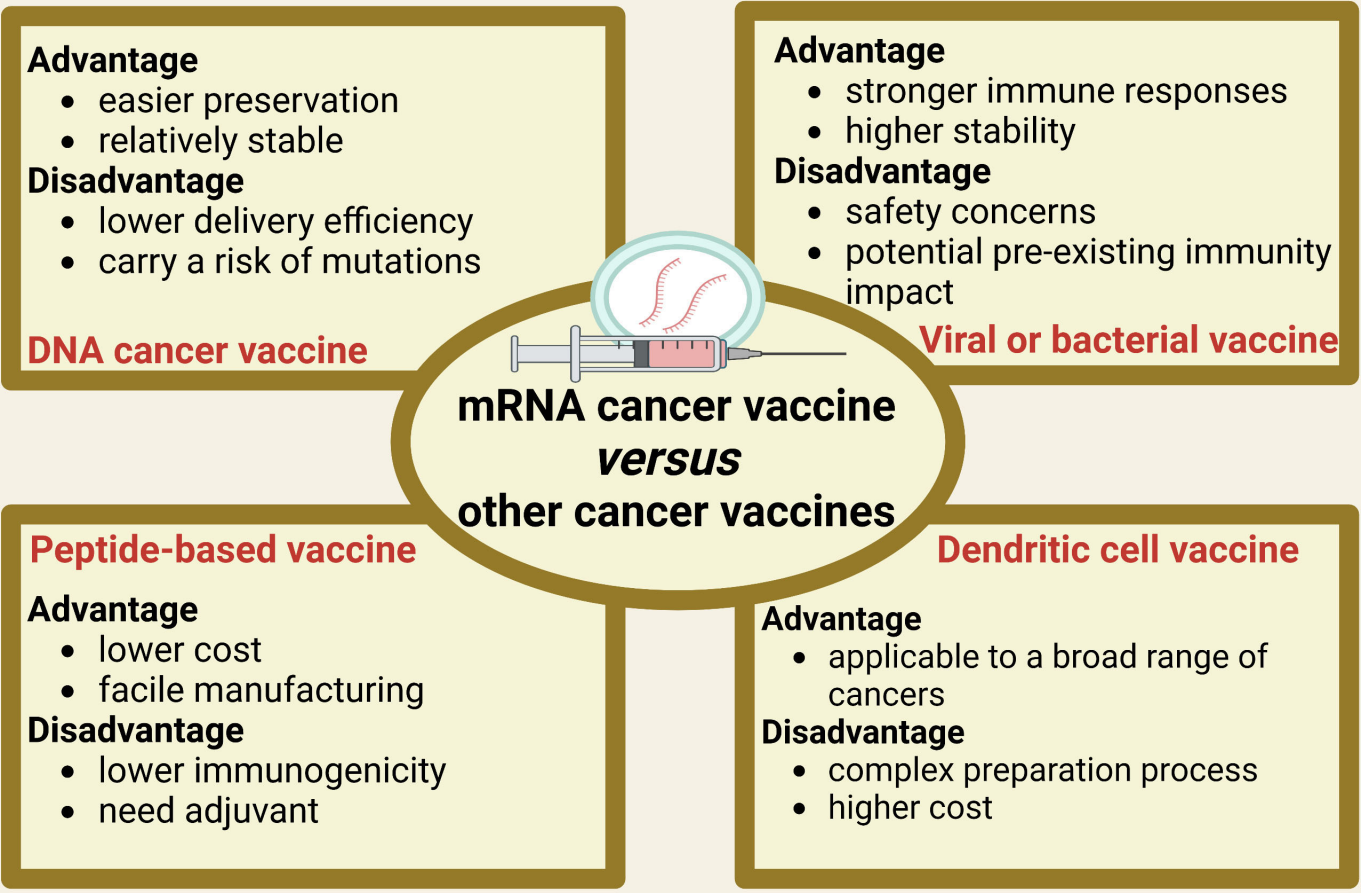
The advantages and disadvantages of other types of cancer vaccines in comparison to mRNA cancer vaccine.

**Figure 2** outlines the advantages and disadvantages of other types of cancer vaccines in comparison to mRNA cancer vaccine. DNA vaccines have certain advantages over mRNA vaccines in terms of storage and stability (28). DNA vaccines can be stored for a long time under regular freezing conditions and are relatively stable, with less degradation (28). However, DNA vaccines also have some drawbacks. DNA vaccines carry a potential risk of integration into the host genome, which may result in insertional mutagenesis (29). Additionally, DNA vaccines have relatively poorer immunogenicity, partly due to their inefficient delivery strategy (29). Compared to mRNA cancer vaccines, bacterial and viral vector vaccines elicit stronger immune responses and exhibit high stability under different storage conditions (15, 30). However, they also face drawbacks such as safety concerns associated with live vectors and potential impact of pre-existing immunity on effectiveness (15, 30). Peptide-based cancer vaccines offer advantages in terms of facile manufacturing and lower cost compared to mRNA cancer vaccines (31). However, they are inferior to mRNA cancer vaccines in terms of immunogenicity, which may result in relatively weaker vaccine efficacy, necessitating the implementation of additional measures to enhance immune response (32). Adjuvants are typically required to enhance immune response for peptide-based vaccines (31, 32). Compared to mRNA cancer vaccine, dendritic cell (DC) vaccines have an advantage because DC cells comprise a versatile cell type capable of engaging multiple facets of the immune system, making them more applicable to a broad range of cancers (33). However, the production of DC cancer vaccines requires complex operational steps and higher costs than mRNA cancer vaccine (33). Despite the inherent advantages and disadvantages of different types of cancer vaccines, mRNA cancer vaccines exhibit a distinctive amalgamation of characteristics that hold great promise for revolutionizing cancer treatment. However, to fully harness their potential, extensive research and clinical trials are required to optimize the efficacy, safety, and implementation of mRNA cancer vaccines.

## 3 Classification and mechanism of action mRNA cancer vaccines

**Figure 3:**
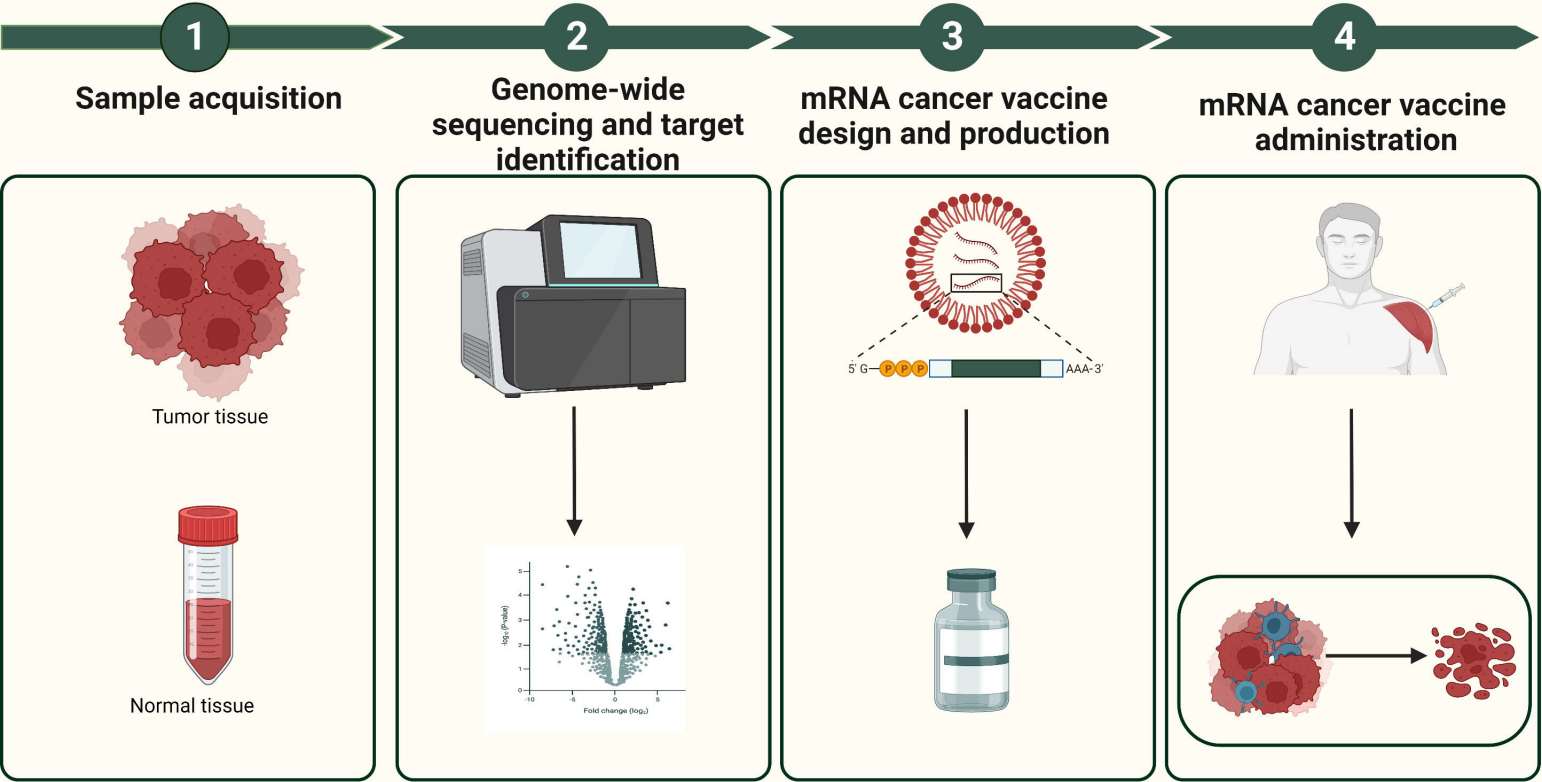
The design and production process of mRNA cancer vaccines. The design and production process of mRNA cancer vaccines include sample acquisition, gene sequencing and target identification, mRNA sequence design, vaccine production, as well as administration of the final vaccine product.

**Figure 4:**
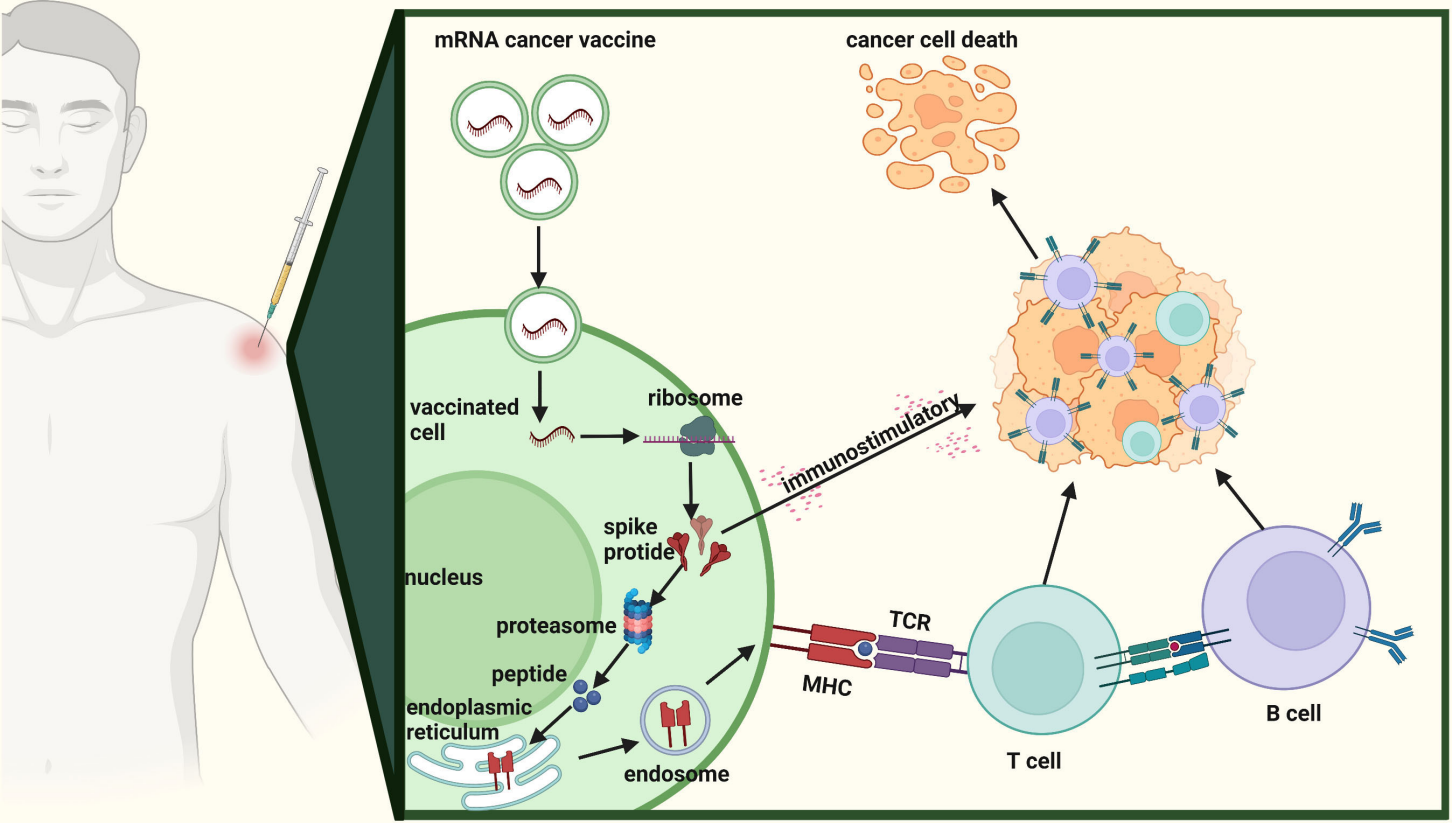
Mechanism of action of mRNA cancer vaccines. The mechanism of action of mRNA cancer vaccines involving internalization of the vaccine into cells, transcription of mRNA sequences encoding selected targets, delivery to immune cells, stimulation of the immune system, and promotion of tumor cell killing.

mRNA cancer vaccines hold immense potential as a personalized cancer therapy that harnesses the patient’s own immune system to specifically target and eliminate tumor cells (12). The progress in next-generation sequencing technologies has made it possible to quickly and cost-effectively compare tumor and normal sequences, serving as the initial stage for identifying cancer targets (34). **Figure 3** shows the comprehensive design and production process of mRNA cancer vaccines, which encompasses sample acquisition, gene sequencing and target identification, mRNA sequence design, vaccine production, as well as administration of the final vaccine product. These vaccines can be categorized into three distinct types - those encoding TSAs, TAAs, and immunostimulatory factors– with each type involving internalization of the vaccine into cells, transcription of mRNA sequences encoding selected targets, delivery to immune cells, stimulation of the immune system, and promotion of tumor cell killing (**Figure 4**) (13, 35, 36). Each type of vaccine has a unique mechanism of action, characteristics, and potential advantages for cancer treatment.

mRNA cancer vaccines encoding TSAs constitute a promising form of personalized cancer therapy, designed to selectively target and eliminate tumor cells while minimizing damage to healthy cells (37). The presence of TSAs is restricted to tumor cells, thereby supporting the generation of specific immune responses against individual tumor antigens in patients (38). This specificity grants mRNA cancer vaccines encoding TSAs several advantages over traditional anti-cancer therapy, including greater efficacy and reduced toxicity (39). Furthermore, these vaccines are highly immunogenic and effective in stimulating robust T cell responses, as the selected targets are specific to the tumor and less likely to have been eliminated during the development of immune self-tolerance (40). Notably, mRNA cancer vaccines encoding TSAs can be designed to target various types of mutations, including single nucleotide variants and indels resulting in frameshift mutations that may alter protein function. Once synthesized, these mRNA sequences are delivered to the patient’s immune cells either *in vitro* or *in vivo* using lipid nanoparticle delivery systems or electroporation techniques (41–43). This ensures that the patient’s immune cells are equipped with the necessary tools to identify and destroy cancer cells.

TAAs, which include cancer or germline genes and lineage-specific differentiation markers, have emerged as attractive targets for vaccine development (26). Among the promising strategies in cancer immunotherapy are mRNA cancer vaccines encoding TAAs (44). These vaccines are designed to stimulate an immune response against proteins expressed by both cancer cells and normal cells (13). The principle behind mRNA cancer vaccines encoding TAAs is to induce a specific immune response against antigens that are overexpressed on the surface of cancer cells relative to normal cells. The process involves identifying the TAAs expressed by the patient’s tumor cells and designing mRNA sequences that encode those antigens. Subsequently, the mRNA is delivered into immune cells, translated and processed into antigen-major histocombatibility complex (MHC) class I/II complexes and then presented on the surface of these cells. This presentation triggers an immune response specifically targeting cells expressing these antigens. Importantly, mRNA cancer vaccines encoding TAAs offer significant advantages, especially in treating certain types of cancer such as lung cancer or melanoma, which exhibit elevated levels of TAAs expression (13).

Immune-regulating factors are molecules capable of either stimulating or suppressing specific immune cell functions, inclusive of cytokines, co-stimulatory ligands and receptors (45). The mRNA-encoded immunostimulatory factors vaccine represents a novel class of vaccines that utilizes mRNA as a vehicle for encoding immune-regulating factors, ultimately augmenting the immune response against cancer cells. The fundamental principle that underlies the design of this vaccine is to increase the concentration of immune-regulating factors, thereby promoting immune system function and bolstering defense against cancer cells. The mRNA vaccine encoding immune stimulatory factors can simultaneously introduce encoded sequences of multiple immune stimulatory factors, activating and enhancing immune system responses through various pathways. In recent studies, the most commonly used cytokines include IL-2, IL-12 and OX40L (46). These cytokines have been shown to stimulate T-cell proliferation and enhance the anti-tumor immune response (47–49). Furthermore, the mRNA vaccine encoding immune stimulatory factors can serve as an adjuvant for the mRNA vaccine encoding TAAs. By using the mRNA encoding TAAs together with the mRNA encoding immune stimulatory factors, the immune activity and anti-tumor effects of the TAAs vaccine can be enhanced. The presence of immune stimulatory factors can augment the immune response elicited by the mRNA encoding TAAs, further facilitating effective recognition and targeting of tumor cells by the immune system. A recent prominent vaccine of interest is ECI-006, which is a combination mRNA cancer vaccine comprising TriMix (mRNA encoding DC activation molecules CD40L, CD70, and caTLR4) and mRNA encoding TAAs (tyrosinase, gp100, MAGE A3, MAGE C2, and PRAME). This innovative vaccine holds promise in boosting the immune response against cancer cells, making it a significant focus of current research.

## 4 Clinical trials of mRNA cancer vaccines

Both preclinical and clinical evidence have shown that the use of mRNA for prophylaxis and therapy has potential in preventing infectious diseases and treating cancers (50). Additionally, mRNA vaccines have demonstrated a safe and well-tolerated profile in both animal models and human trials (51). As of April 30th, 2023, ClinicalTrials.gov reports that 35 clinical trials are actively assessing the safety and efficacy of mRNA cancer vaccines for select cancer types (**Table 1**). These trials comprise preliminary investigations into the pharmacological, dosing, and immunogenic features of mRNA vaccines, as well as larger-scale evaluations of their potential to mitigate tumor recurrence or enhance survival rates. However, it is noteworthy that these studies remain in their initial phases, and additional research and validation will be crucial to corroborate their effectiveness.

**Table 1:** Characteristics of ClinicalTrials.gov registered mRNA cancer vaccines, including those encoding TSAs, TAAs, and immunostimulatory factors.

NCT member	Vaccine type	Target antigen	Sponsor	Cancer type	Vaccine routes	Phase	Combination
mRNA cancer vaccines encoding TSAs							
NCT03639714	GRT-C901/GRT-R902	Personalized neoantigen	Gritstone bio	NSCLC; Colorectal cancer; Gastroesophageal Adenocarcinoma; Urothelial Carcinoma	IM	I; II	Ipilimumab; Nivolumab
NCT03897881	mRNA-4157	Up to 34 neoantigen	Moderna	Melanoma	IM	II	Pembrolizumab
NCT03313778	mRNA-4157	Several neoantigen	Moderna	Unresectable solid tumors	IM	I	Pembrolizumab
NCT03480152	mRNA-4650	Up to 20 neoantigen	National Cancer Institute	Solid Tumors	IM	I; II	None
NCT03289962	Autogene Cevumeran (BNT 122)	Up to 20 neoantigen	Genentech	Melanoma; NSCLC; Bladder ; Colorectal; Triple Negative Breast; Renal; Head and Neck; Other Solid Cancers	IVI	I	Atezolizumab
NCT03908671	Personalized mRNA Tumor Vaccine	NA	Stemirna Therapeutics	Advanced esophageal cancer and NSCLC	SC	NA	None
NCT03468244	Personalized mRNA Tumor Vaccine	NA	Changhai Hospital	Solid Tumors	SC	NA	None
NCT05761717	Personalized mRNA Tumor Vaccine	NA	Shanghai Zhongshan Hospital	Postoperative Hepatocellular Carcinoma	SC	NA	Stintilimab
NCT04486378	RO7198457	Encoding individual mutations	BioNTech	Colorectal Cancer	IVI	II	None
NCT04161755	RO7198457	Encoding individual mutations	BioNTech	Pancreatic Cancer	IVI	I	Atezolizumab
NCT03815058	RO7198457	Encoding individual mutations	Genentech	Advanced melanoma	IVI	II	Pembrolizumab
NCT03289962	RO7198457	Encoding individual mutations	Genentech	Solid tumors	IVI	I	Atezolizumab
NCT02316457	VAC_W_bre1_uID and IVAC_M_uID	BC TAA and encoding individual mutations	BioNTech	TNBC	IVI	I	None
mRNA cancer vaccines encoding TAAs							
NCT03948763	mRNA-5671/V941	KRAS G12D/G12V/G13D/G12C	Merck Sharp & Dohme LLC	KRAS Mutant NSCLC and Colorectal cancer and Pancreatic Adenocarcinoma	IVI	I	Pembrolizumab
NCT04526899	BNT111	NY-ESO-1, tyrosinase, TPTE and MAGE-A3	BioNTech	Melanoma	IVI	II	Cemiplimab
NCT02410733	Lipo-MERIT(BNT111)	As above	BioNTech	Advanced melanoma	IVI	I	None
NCT04382898	BNT112	PAP, PSA, and three undisclosed antigens	BioNTech	Prostate Cancer	IVI	I; II	Cemiplimab
NCT04534205	BNT113	HPV16 E6 and E7 oncoproteins	BioNTech	Head and neck squamous cell carcinoma	IVI	II	Pembrolizumab
NCT03418480	BNT113	HPV16 E6 and E7 oncoproteins	University of Southampton	Advanced HPV16+ cancer	ID	I; II	Anti-CD40 antibodies
NCT04163094	BNT115	Ovarian cancer TAA	University Medical Center Groningen	Ovarian cancer	IVI	I	carboplatin plus paclitaxel
NCT05142189	BNT116	NSCLC TSA	BioNTech	NSCLC	IVI	I	Cemiplimab plus Docetaxel
NCT05557591	BNT116	NSCLC TSA	BioNTech	Advanced NSCLC with PDL1 ≥ 50%	IVI	I; II	Cemiplimab
NCT05714748	EBV mRNA vaccine	EBV oncoproteins	West China Hospital	EBV-positive advanced malignant Tumors	IM	I	None
NCT03164772	BI 1361849	MUC1, survivin, NY-ESO-1, 5T4, MAGE-C2, and MAGE-C1	Ludwig Institute for Cancer Research	Metastatic Non-small Cell Lung Cancer	ID	I; II	Durvalumab, Tremelimumab
NCT05738447	HBV mRNA vacccine	HBV oncoproteins	West China Hospital	HBV-related Refractory Hepatocellular Carcinoma	IM	I	None
NCT04573140	RNA-LP vaccine	Tumor mRNA and pp65 LAMP	University of Florida	Glioblastoma	IVI	I	None
NCT04503278	CARVac	Encoding CLDN6	BioNTech Cell & Gene Therapies GmbH	CLDN6-positive relapsed or refractory advanced solid tumors	IVI	I; II	CAR-T therapy
NCT03394937	ECI-006	TAA: gp100, tyrosinase, MAGE-A3, MAGE-C2, PRAME	eTheRNA immunotherapies	Melanoma	Intranodal	I	None
mRNA cancer vaccines encoding immunostimulatory factors							
NCT03871348	SAR441000(BNT 131)	IL-12sc, IL-15 sushi, GMCSF, IFNa	Sanofi	Metastatic Neoplasm	intratumorally	II	Cemiplimab
NCT04455620	BNT151	Optimized IL-2	BioNTech	Solid Tumor	IVI	I; II	
NCT04710043	BNT152+153	IL-7, IL-2	BioNTech	Solid Tumor	IVI	I	None
NCT03291002	CV8102	TLR7/8, RIG-1	CureVac	Skin cancer	intratumoral	I	anti-PD-1
NCT03323398	mRNA-2416	OX40L	Moderna	Relapsed/Refractory Solid Tumor Malignancies	intratumoral	I; II	Durvalumab
NCT03739931	mRNA-2752	OX40L, IL-23, IL-36g	Moderna	Relapsed/Refractory Solid Tumor Malignancies or Lymphoma	intratumoral	I	Durvalumab
NCT03946800	MEDI1191	IL-12	MedImmune LLC	Solid Tumors Cancer	intratumoral	I	Durvalumab

TSAs, tumour-associated antigens; TAAs, tumor associated antigens; LAMP, lysosomal associated membrane protein; TNBC, triple negative breast cancer; BC, breast cancer; EBV, epstein-barr virus; HBV, hepatitis B virus; NY-ESO-1, New York esophageal squamous cell carcinoma 1; MAGE-A3, melanoma-associated antigen A3 tyrosinase; TPTE, transmembrane phosphatase with tensin homology; IM ,intramuscular; IVI, intravenous injection; SC, subcutaneous injection; ID, intradermal administrations; NA, Not applicable.

### 4.1 mRNA cancer vaccines encoding TSAs

**Table 1** presents a summary of registered clinical trials based on clinicaltrials.gov for mRNA cancer vaccines encoding TSAs. One of these vaccines, mRNA-4157, is a personalized mRNA vaccine that has the capacity to encode up to 34 antigens and is currently being assessed for its effectiveness in treating melanoma (NCT03897881). Results from the phase IIb KEYNOTE-942 trial indicate that mRNA-4157/V940 combined with pembrolizumab could be a potential adjuvant therapy for melanoma, as patients receiving this combination had a significant reduction in disease recurrence risk compared to those who only received PD-1 inhibitor, showing promising results (52). In a phase 1 clinical trial, autogene cevumeran (BNT 122) containing up to 20 neoantigens was tested for its ability to stimulate immunity against neoantigens in patients with resected pancreas ductal adenocarcinoma (PDAC). The results revealed that patients who responded to the vaccine exhibited a longer recurrence-free survival than non-responders at an early median follow-up of 15 months, suggesting that vaccine-induced neoantigen-specific immunity may be associated with improved outcomes in PDAC (53). However, evidence for the effectiveness of mRNA-4650 in treating gastrointestinal cancer appears to be discouraging. A study by Cafri et al. evaluated the immunogenicity and clinical efficacy of mRNA-4650 and only observed an increase in the frequency of cancer-specific T cells, but no clinical benefit (22).

Currently, several institutions such as BioNTech and Moderna have joined forces in the pursuit of developing personalized mRNA cancer vaccines, with numerous clinical trials currently underway. Despite having a theoretically proven efficacy, these vaccines face obstacles in areas such as design, production, and cost. Thus, additional optimization efforts are necessary to overcome these challenges. Furthermore, further preclinical and clinical trials are indispensable in validating the effectiveness of personalized mRNA cancer vaccines. These trials can identify opportunities for improvement and expedite the translation of this innovative approach from bench to bedside, ultimately benefiting cancer patients.

### 4.2 mRNA cancer vaccines encoding TAAs

Fifteen clinical trials are currently in progress to assess mRNA cancer vaccines that encode TAAs, as demonstrated in **Table 1**. The BNT111 vaccine developed by BioNTech is an example of an mRNA cancer vaccine that encodes four melanoma-associated antigens (MAAs) including New York esophageal squamous cell carcinoma 1 (NY-ESO-1), melanoma-associated antigen A3 (MAGE-A3), tyrosinase, and transmembrane phosphatase with tensin homology (TPTE) (44). These RNAs are encapsulated in liposomes and administered intravenously to patients. In a phase I trial, this vaccine alone and in combination with ICIs induced durable objective responses and had a favorable safety profile among patients with advanced melanoma (44). Currently, a phase II trial is underway evaluating the vaccine candidate in combination with the anti-PD-1 antibody cemiplimab for patients with unresectable stage III or stage IV melanoma who are refractory to or have relapsed after anti-PD-1 therapy. These findings suggest that the BNT111 vaccine holds great promise as a treatment option for melanoma and may provide new hope for patients with advanced forms of the disease.

mRNA-5671 is a tetra-valent vaccine that has been formulated with lipid nanoparticle (LNP) technology and is based on messenger RNA (36). This innovative vaccine targets four of the most frequent KRAS mutations - G12D, G13D, G12C, and G12V (36). Pre-clinical investigations have indicated a substantial improvement in CD8 T cell responses to KRAS antigens post-immunization with mRNA encoding KRAS mutations (36). In a phase I trial, patients suffering from advanced or metastatic NSCLC, colorectal cancer, or pancreatic adenocarcinoma, and having KRAS mutations, are being enrolled to determine the efficacy of mRNA-5671 with or without pembrolizumab (NCT03948763).

A phase 1/2 clinical trial (NCT04382898) is currently underway to evaluate the effectiveness of the cancer vaccine BNT112. This vaccine encodes five different tumour-associated antigens and is being administered alone or in combination with cemiplimab to patients with metastatic castration-resistant prostate cancer. Another trial is a randomised phase 2 study (NCT04534205) evaluating the anti-human papillomavirus (HPV)-16-derived oncoprotein-encoding mRNA BNT113 vaccine in HPV16-positive, PD-L1-positive head and neck squamous cell carcinoma. BNT113 is also being tested in a two-arm phase 1/2 vaccine dose-escalation study (NCT03418480) for patients with previously treated or advanced HPV16-positive head and neck squamous cell carcinoma. Another phase 1 study (NCT04163094) is being conducted to evaluate the BNT115 that encodes ovarian-specific tumour-associated antigens. This vaccine is being administered both before and in combination with adjuvant and neoadjuvant chemotherapies to patients with ovarian cancer. Finally, a fifth clinical trial (NCT05142189) is evaluating the FixVac vaccine BNT116 in combination with cemiplimab or docetaxel in a phase 1 study for patients with advanced or metastatic non-small cell lung cancer.

### 4.3 mRNA cancer vaccines encoding immunostimulatory factors

The profound potential of mRNA cancer vaccines stems from their ability to encode a wide range of proteins, including immunostimulants that can modify the tumor immune microenvironment (TME) and enhance the efficacy of immune checkpoint inhibitors (51). This promising avenue of mRNA vaccine research has spurred clinical trials of cytokine-encoding mRNA products by BioNTech and Moderna, with highly encouraging results (13, 54). For instance, intratumorally administered mRNA-2416 produced by Moderna, which encodes OX40L, demonstrated safety and tolerability in a phase I trial and elicited broad proinflammatory activity with desirable changes in the TME (54). These findings provide critical support for its further investigation in combination therapy with anti-PD-L1 inhibitor durvalumab in solid tumors. Similarly, co-administration of mRNA-2752 encoding OX40L/IL23/IL36g with durvalumab in a dose escalation study (NCT03739931) exhibited antitumor effects, validating the potential of mRNA cancer vaccines as a therapeutic modality. Furthermore, BioNTech’s BNT131 (SAR441000), which encodes IL-12sc, IL-15sushi, IFN-α for intratumoral injection, is being tested as monotherapy and in combination with cemiplimab for patients with advanced solid tumors to alter the TME. Several other mRNA products have also shown promise, such as ECI-006, a combination of TriMix and melanoma-specific TAAs administered intranodularly and being tested in a phase 1 study of resected melanoma (NCT03394937); and MEDI1191, an immunomodulatory fusion protein containing IL-12α and IL-12β subunits developed for intratumoral injection (55). While only seven product candidates are currently undergoing clinical trials, the results thus far demonstrate the immense potential of mRNA cancer vaccines in improving cancer immunotherapy outcomes (**Table 1**). With these promising results, further research is needed to determine the optimal administration route and maximize vaccine efficacy.

## 5 Current challenges and future perspectives

Numerous clinical trials are currently under development or in progress to assess the safety and efficacy of mRNA cancer vaccines (51). Despite promising results in preclinical and early-phase clinical trials, the successful translation of mRNA cancer vaccines into clinical practice faces several obstacles. These challenges include tumor heterogeneity, an immunosuppressive tumor microenvironment, optimal vaccine administration routes, and the identification of biomarkers to monitor treatment response.

### 5.1 Tumor heterogeneity

The complexity of treatment decisions is enhanced by tumour heterogeneity, which can be divided into spatial and temporal heterogeneity (56). Temporal heterogeneity refers to the dynamic evolution of the genomes through the tumour progression course, whereas spatial heterogeneity refers to the phenomenon that a tumour is composed of subclones of different genetic backgrounds (57–59). This heterogeneity leads to variable responses to therapies among individuals with cancers of the same tumour subtype and is believed to be one of the major causes of progressive disease and failure of therapy (60, 61). Typically, only a small fraction of a specimen is sampled for mRNA cancer vaccine design, which is unlikely to provide full information on tumour gene profile, contributing to increased uncertainties for the clinical efficacy of mRNA cancer vaccine (11). Thus, tumour heterogeneity largely limits the efficacy of mRNA cancer vaccine and how to overcome tumor heterogeneity is a difficult challenge for its clinical application.

To overcome spatial heterogeneity in tumours, one strategy is to use tumour tissue multipoint sampling to identify differences between tumour regions and inform the design of personalised mRNA cancer vaccines. Another approach involves using mRNA cancer vaccines that target multiple antigens expressed across various tumour regions, thereby compensating for spatial heterogeneity. Meanwhile, monitoring disease progression and adjusting treatment plans accordingly may help to address temporal heterogeneity. However, these strategies not only increase the complexity of vaccine design and administration but also raise costs and increase treatment time for patients. Another promising method is the use of artificial intelligence algorithms such as MHC-binding prediction, quantification of mutated transcript expression, and clonality of the mutation to predict neoantigens based on tumour genomic data, then used to prioritize these mutations as vaccine candidates based on their likelihood to elicit a T cell response, which could improve the efficiency and accuracy of vaccine design and overcome the heterogeneity of tumour (62–64). However, to date, there is still limited information on artificial intelligence for mRNA cancer vaccine design. This is certainly an important and interesting area worthy of future investigations.

### 5.2 Immunosuppressive tumor microenvironment

Tumour initiation, progression and maintenance depend highly on interactions between the tumour and the associated microenvironment (65). Tumour microenvironment refers to the surrounding microenvironment of tumour cells, including surrounding cells, signalling molecules, and extracellular matrix (66). The importance of the tumor microenvironment in cancer has been recognized since the late 1800s and then accumulating evidences suggested immunosuppressive TME not only promote immune evasion and tumor growth, but also lead to decrease the efficacy of immunotherapy (67–69). Immunosuppressive TME could decrease the efficacy of immunotherapy by inhibiting the function and activation of immune cells, such as T cells and natural killer cells, which are critical for attacking and eliminating cancer cells (14). Although mRNA cancer vaccine may have the capability to elicit a cellular immune response, the inhibitory tumor microenvironment can impede T cell infiltration into tumors and result in T cell exhaustion (50). As with many other immunotherapies, overcoming immunosuppressive TME is one of the most challenging and unsolved problems for mRNA cancer vaccine.

Combining mRNA cancer vaccines with other anti-cancer treatments, which is being constantly tried in cancer patients, can be an effective strategy for overcoming the immunosuppressive microenvironment. One commonly used combination therapy is to combine mRNA vaccines with immune checkpoint inhibitors, which can “release the brakes” on the immune system, allowing it to attack cancer cells more effectively (55, 70). Adoptive T-cell therapy, another type of immunotherapy, can also be used alongside mRNA vaccines. This innovative treatment involves extracting T-cells from a patient’s blood or tumor, modifying them in a laboratory to target specific cancer antigens, and then reintroducing them into the patient’s body. Combining these modified T-cells with mRNA vaccines can significantly enhance the immune response against cancer cells, thus improving the efficacy of this therapy (71). The combination of mRNA vaccines and radiotherapy has also shown promising therapeutic effects in preclinical and clinical models (72, 73). Overall, combining mRNA cancer vaccines with other anti-cancer treatments can be a powerful approach to overcoming the immunosuppressive microenvironment in tumors. Continued research and development of these combination therapies will be critical to improving outcomes for cancer patients.

### 5.3 Vaccine administration routes

**Figure 5:**
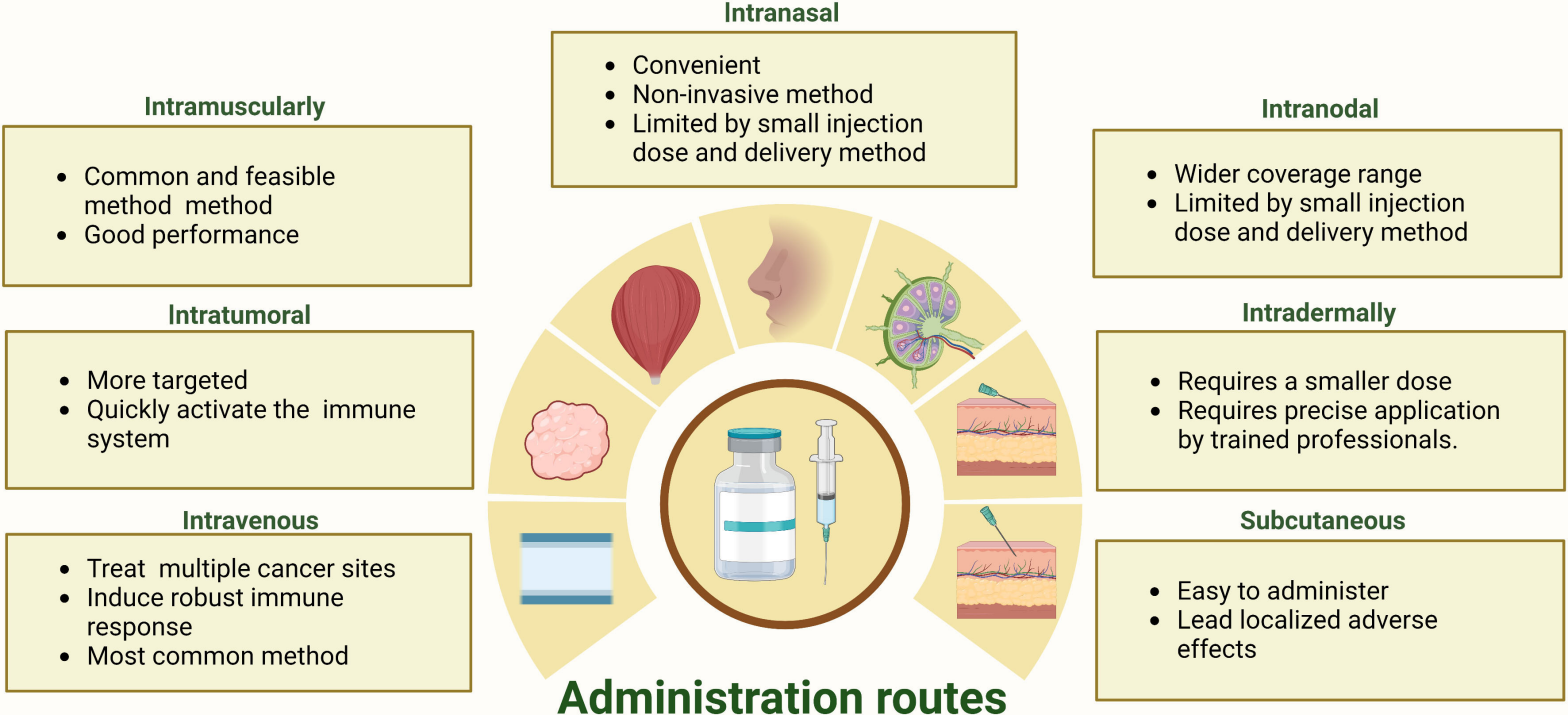
The strengths and limitations inherent in a range of administration routes for mRNA cancer vaccines.

Poor performance in any step of the mRNA delivery process would compromise the therapeutic efficacy, and administration routes should be a first-order consideration for the clinical usage of mRNA cancer vaccine (74, 75). The administration route of mRNA vaccines strongly influences the translation efficiency of the target protein and the distribution of mRNA cancer vaccine *in vivo (* 16, 76). Currently, there is no consensus on the optimal route of administration for mRNA vaccines, although the SARS-CoV-2 mRNA vaccines that have received approval utilize intramuscular injection (50). Various routes, including intramuscular, subcutaneous, intranodal, intradermal, and intranasal vaccination, are actively being explored in the search for an optimal administration route for mRNA cancer vaccines (55). Each administration route for mRNA cancer vaccines has unique advantages and limitations. As depicted in **Figure 5**, the strengths and weaknesses of diverse mRNA cancer vaccines administration routes are presented.

Intramuscular and intravenous injections are the two most frequently used administration routes in clinical trials for mRNA cancer vaccines (**Table 1**). Intramuscular administration is a widely used and feasible vaccination route that involves the direct injection of a vaccine into muscle tissue (14, 77). This method has demonstrated efficacy in inducing an immune response for mRNA cancer vaccines, being both easily executed and well-tolerated with the added benefit of offering flexible dosing options, while also causing minimal side effects at the injection site (78). Consequently, its use has become prevalent (14, 77, 78). Additionally, intravenous injection is the most frequently used direct administration route in current clinical trials for active therapeutic mRNA cancer vaccines (79). It has been shown to be safe, well-tolerated, and capable of inducing an immune response against cancer cells (80). Furthermore, it enables the vaccine to reach multiple lymphoid organs and allows for repeated dosing to maintain immunity over time (55, 80).

Intranasal administration of mRNA cancer vaccines can effectively deliver the vaccine to antigen-presenting cells in the peripheral lymph nodes (81, 82). However, intranodal injections provide a more direct route to reach lymphatic antigen-presenting cells (83). While both delivery methods have their advantages, they also face significant limitations. Intranasal administration may offer non-invasive delivery but is restricted by nasal cavity volume constraints (81, 82). Conversely, intranodal injections require specialized equipment and highly skilled personnel, and the injection volumes are small, which may not be sufficient for larger tumors or inducing a robust immune response (84). Accordingly, further research is necessary to optimize these methods for mRNA cancer vaccines.

Intratumoral injections of mRNA cancer vaccines, which can rapidly activate immune cells and minimize off-target, have been investigated in clinical and preclinical trials (85, 86). Although this approach aims to induce local inflammation with mRNA encoding immunostimulatory, its effectiveness is limited by the size and location of the tumor, which may restrict the amount of vaccine that can be delivered (87). Repeated injections may be required to maintain the immune response over time, and combining intratumoral injections with systemic delivery methods such as immune checkpoint inhibitors may offer a more comprehensive approach to treating cancer (55). Despite these limitations, intratumoral injections remain a promising area of research for directly interact with immune cells to activate an immune response mRNA cancer vaccine. Intradermal and subcutaneous injections are two common administration routes for mRNA cancer vaccines (74). Both methods allow regional antigen-presenting cells to easily process the mRNA, which is essential for eliciting an immune response against cancer cells (74). However, these administration routes often induce severe local injection-site reactions than intramuscular administration, which can negatively impact patient compliance and overall treatment efficacy (14, 74).

In view of the immense potential offered by various routes of administration for successful cancer therapy, it is of paramount importance to thoroughly assess the advantages and disadvantages of each vaccination route. Recently, investigators have utilized a non-invasive method involving a dual radionuclide near-infrared probe to track the spatiotemporal trafficking of the vaccine following intramuscular injection, offering essential guidance in precisely evaluating the dose, injection site, and biological distribution of the vaccine (88). In order to determine the most effective vaccination route and optimize vaccine efficacy, there remains a pressing need for novel approaches that can accurately monitor and analyze the spatiotemporal kinetics of vaccines (88). By carefully weighing the pros and cons of each administration route, we can identify the most promising strategies for delivering immunotherapies that can target cancer cells effectively while minimizing off-target effects. Furthermore, by leveraging advanced tools to track and analyze vaccine movement within the body, we can acquire critical insights into the factors that govern their biological activity and efficacy. Therefore, these innovative methods hold tremendous promise for advancing our understanding of how to optimize mRNA cancer vaccination routes and maximize the impact of cancer immunotherapy.

### 5.4 Biomarkers for monitoring the treatment response

The introduction of mRNA cancer vaccines represents an exciting new frontier in cancer treatment, but also underscores the need for novel efficacy evaluation standards. Unlike traditional cancer treatments, such as chemotherapy, targeted therapy, and radiation therapy, which targeting the tumor cells directly and emphasizing tumor shrinkage as a successful response to treatment evaluated by radiological imaging, mRNA vaccines stimulate the immune system to produce an anti-tumor response indirectly that standard radiographic imaging techniques do not account for this unique mechanism of action and may not reflect the true clinical benefit of mRNA vaccines (89). mRNA cancer vaccines may lead to inflammatory reactions resulting in tumour swelling, which results in increased complexity of evaluating the efficacy of mRNA cancer vaccines (90). Moreover, cancer vaccines, particularly when used as monotherapy, may exhibit greater efficacy in cases of low disease burden and may not produce the striking radiographic responses typically observed with cytotoxic therapy (91). As a result, the development of cancer vaccines may encounter difficulties in demonstrating effectiveness when evaluated in late-stage disease using traditional assessment methods such as standard radiographic response evaluation criteria (92). Due to their highly individualized and customized nature, personalized mRNA cancer vaccines require different treatment plans and dosages tailored to each patient’s unique condition and cancer type (89). To meaningfully identify which mRNA cancer vaccines should advance beyond early phase trials and into larger phase III clinical trials, novel biomarkers need to establish that can accurately monitor the treatment response to these vaccines.

Quantitative measurement of immune cell responses is essential for establishing surrogate biomarkers of efficacy, especially for tumor antigen-specific T cell responses, which are critical in selecting optimal doses for cancer vaccine trials and have the potential to lead to tumor rejection. Multiple studies are evaluating the effectiveness of mRNA therapies by measuring immune response indicators, such as cytokines, chemokines, or immune cells in the blood. A variety of immune monitoring techniques, including flow cytometry analysis of cell populations’ phenotypes, functionalities, and activation status, are employed (93). Additionally, enzyme-linked immunospot (ELISPOT) assays measure cytokine release after antigen-specific immune responses, while peripheral cytokine profiling using enzyme-linked immunosorbent assay evaluates innate immune responses (93–95). Another method, tetramer analysis with MHC multimers loaded with antigen peptides, is used to measure antigen-specific CD8 T cells, and T cell receptor analysis through sequencing and polymerase chain reaction helps to elucidate the immune repertoire, including genetic arrangement and specificity. These assays are applied to immune cells in peripheral blood mononuclear cells acquired from patients to detect and describe T cell responses. Although ELISPOT is one of the most commonly used techniques to identify CD8 T cell responses to a given antigen, its quantitative output cannot determine the absolute number of antigen-specific T cells (94). It is noteworthy that currently available immunological assays, such as ELISPOT, flow cytometry-based multimer staining, and intracellular cytokine staining, have been found to exhibit technical inconsistencies across different laboratories (96). For example, one study reported inter-laboratory variations of up to 50% in ELISPOT (96). Standardized and harmonized procedures, from specimen banking to assay validation and result reporting, are thus necessary for successful clinical development.

Over the past few decades, single-cell RNA sequencing (scRNA-seq) technology has garnered significant attention as a novel tool in assessing the immunotherapy response of cancer patients (97). The scRNA-seq technology refers to a collection of techniques that enable the untargeted quantification of transcripts present in individual cells, facilitating the identification of cell types and states associated with immunotherapy response (97, 98). The scRNA-seq technology allows for the comprehensive analysis of cellular transcriptomes, facilitating the identification of distinct immune cell subsets and their gene expression patterns correlated with immunotherapy response (99). Furthermore, this technique provides insights into the dynamic changes within the tumor microenvironment during immunotherapy, aiding in the understanding of mechanisms underlying treatment response (99). Recently, multiple studies have reported utilized scRNA-seq to explore immune cell subset infiltration and regulation changes pre- and post-mRNA vaccine administration to understand therapeutic response and analyze underlying mechanisms to determine mRNA vaccine efficacy. With further advancements in technology and deeper applications, scRNA-seq technology holds the promise of providing valuable insights into mRNA cancer vaccine efficacy detection.

Recently, the circulating tumour DNA (ctDNA) test has the advantages of sensitivity, flexibility, repeatability, and safe gain much attention to help the evaluation of treatment responses, and the application of ctDNA detection in monitoring mRNA cancer vaccine therapeutic response is a worthwhile attempt (100). Accumulating evidence suggests that changes in ctDNA levels during treatment compared to baseline can serve as a valuable biomarker for stratifying patients as molecular responders or nonresponders and distinguishing those with a favorable prognosis, especially those displaying stable disease according to RECIST v1.1 criteria (101). Palmer et al. investigated the utility of ctDNA as a potential biomarker for monitoring therapeutic responses to mRNA cancer vaccines (70). Their study demonstrated that a decrease in ctDNA levels among patients undergoing treatment was positively correlated with prolonged overall survival, thereby underscoring ctDNA’s potential as a promising biomarker for predicting treatment response (70). The future exploitation of efficacy evaluation system integration of immunologic and radiologic endpoints, the establishment of biomarkers, and standardization of evaluation protocols is needed to develop in the future, which will be necessary to realize the full potential of this promising technology in the fight against cancer.

## 6 Conclusions

Altogether, mRNA cancer vaccines present a promising new approach to anticancer therapies with both opportunities and challenges. The highly personalized and specific nature of this technology offers tremendous potential for precision medicine in the fight against cancer. However, further clinical trials are necessary to fully establish the safety and efficacy of mRNA cancer vaccines and additional preclinical studies are warranted to explore the combined use of mRNA cancer vaccine and other anticancer therapies. Additionally, addressing issues such as tumoral heterogeneity, routes of administration and development of methods to assess the efficacy processes will be critical for advancing this technology toward meaningful clinical outcomes. With continued research and investment, mRNA cancer vaccines hold great promise as a transformative therapy for cancer patients.

## Author contributions

BW and JL collected relevant literature and wrote the manuscript. BW and JY conceptualized the main structure of this review, and revised and validated the final version. JP, SX and BW contributed to the literature analysis and manuscript editing. All authors contributed to the article and approved the submitted version.
